# Toward a Diagnostic Score in Cushing's Syndrome

**DOI:** 10.3389/fendo.2019.00766

**Published:** 2019-11-08

**Authors:** Leah T. Braun, Anna Riester, Andrea Oßwald-Kopp, Julia Fazel, German Rubinstein, Martin Bidlingmaier, Felix Beuschlein, Martin Reincke

**Affiliations:** ^1^Department for Endocrinology, Medizinische Klinik und Poliklinik IV, Ludwig-Maximilians-University, Munich, Germany; ^2^Klinik für Endokrinologie, Diabetologie und Klinische Ernährung, Universitätsspital Zürich, Zurich, Switzerland

**Keywords:** Cushing's syndrome, hypercortisolism, diagnostic score, Cushing's disease, low-dose-dexamethasone-suppression-test, urine cortisol

## Abstract

Cushing's syndrome (CS) is a classical rare disease: it is often suspected in patients who do not have the disease; at the same time, it takes a mean of 3 years to diagnose CS in affected individuals. The main reason is the extreme rarity (1–3/million/year) in combination with the lack of a single lead symptom. CS has to be suspected when a combination of signs and symptoms is present, which together make up the characteristic phenotype of cortisol excess. Unusual fat distribution affecting the face, neck, and trunk; skin changes including plethora, acne, hirsutism, livid striae, and easy bruising; and signs of protein catabolism such as thinned and vulnerable skin, osteoporotic fractures, and proximal myopathy indicate the need for biochemical screening for CS. In contrast, common symptoms like hypertension, weight gain, or diabetes also occur quite frequently in the general population and *per se* do not justify biochemical testing. First-line screening tests include urinary free cortisol excretion, dexamethasone suppression testing, and late-night salivary cortisol measurements. All three tests have overall reasonable sensitivity and specificity, and first-line testing should be selected on the basis of the physiologic conditions of the patient, drug intake, and available laboratory quality control measures. Two normal test results usually exclude the presence of CS. Other tests and laboratory parameters like the high-dose dexamethasone suppression test, plasma ACTH, the CRH test, and the bilateral inferior petrosal sinus sampling are not part of the initial biochemical screening. As a general rule, biochemical screening should only be performed if the pre-test probability for CS is reasonably high. This article provides an overview about the current standard in the diagnosis of CS starting with clinical scores and screenings, the clinical signs, relevant differential diagnoses, the first-line biochemical screening, and ending with a few exceptional cases.

## Incidence, Epidemiology, and Time to Diagnose CS

Cushing's syndrome (CS) is a rare disease leading to atypical and rare symptoms for age, but it can be associated with disease features that are quite common in the general population. The incidence of endogenous CS has been estimated to be 0.7–2.4 to 0.2–5.0 per million people per year (1, 2). About 80% of cases are ACTH-dependent, whereas 20% of Cushing cases are of primary adrenal, ACTH-independent origin. ACTH-dependent CS can be further divided into pituitary-dependent CS and ectopic, paraneoplastic CS. While the latter is quite seldom, causing not more than 10% of cases, CRH-producing tumors account for even fewer cases (3). Because of the universal expression of the glucocorticoid receptor, the list of symptoms and clinical signs is long and lacks a clear focus. This, and the overlap with symptoms of the metabolic syndrome, makes diagnostics challenging.

The rareness of the disease, the broad symptomatology without a lead symptom, and the clinical overlap with features of the metabolic syndrome often results in a delayed diagnosis. Generally, it can take up to 4 years and longer from the beginning of disease symptoms to the actual diagnosis (4). On average, 4.6 physicians will have to be consulted according to one study (4). This is hardly acceptable, considering that CS is an endocrinological “emergency” associated with relevant morbidity and mortality. Mortality is highest in patients with severe hypercortisolism and persisting and ectopic CS (5), and a relevant number of patients die before or within 90 days after initiation of treatment (6). The diagnostic delay might be one of the reasons for the long-term negative aspects and comorbidities (7–9) and high morbidity in these patients even after achieving remission of the actual disease. We have recently analyzed in a systematic meta-analysis of 45 studies on 5,560 patients with CS time to diagnosis and found that it has remained unchanged with a mean time of 34 months (10). This clearly argues for considerations beyond established awareness campaigns. New approaches including automated face recognition, easy-to-apply scoring systems, or focused rational diagnostics will be instrumental to overcome the current challenges. In the following, we will give a critical overview of the current standard in the diagnosis of CS, mainly focusing on the prevalence of different clinical symptoms in patients with CS.

## Clinical Reasoning Problems

Solving clinical cases correctly (11)—but also efficiently (12)—is one of the great challenges in daily clinical practice. Diagnostic error rate in internal medicine is generally high (at least 10%) (13) due to a lack of knowledge (14), premature closure, and forgetting to consider other diagnoses (15). As *Clinical Reasoning* is mainly based on pattern recognition and illness script formation (16, 17), it is quite clear that diagnosing CS correctly is even more challenging than many other diseases: statistically, a family practitioner diagnoses between 0 and 1 patient with CS in his work-life. Even endocrinologists seldom diagnose patients with CS, apart from endocrinologists in specialized outpatient clinics. Therefore, as physicians will only evaluate a small number of true Cushing patients, it is difficult for them to develop the corresponding illness scripts to diagnose the disease in time. To foster the clinical reasoning process, different attempts have been made.

## Attempts to Fasten the Diagnostic Process

As the pretest probability is generally low because of the rareness of CS, it is mandatory to preselect patients with an appropriate likelihood of endogenous CS and use laboratory tests with high sensitivity and specificity. Different strategies starting from clinical scores, face recognition, and ending with extended screening have been used in the past decades to improve differential diagnostics:

### Clinical Scores for CS

As early as 1964, Nugent et al. developed a clinical score to identify patients with CS (18) correctly. To simplify the diagnostic process, he used a combination of clinical signs and symptoms to discriminate patients with hypercortisolism from patients without it. The following 19 signs are part of his scoring: osteoporosis, central obesity, generalized obesity (muscle) weakness, plethora, WBC (white blood cells), acne, red and purple striae, diastolic blood pressure, edema, hirsutism, ecchymoses, serum potassium, oligomenorrhea, headaches, MCV, abnormal glucose tolerance, and age. However, only half of the patients with CS can be correctly identified using this scoring, and therefore, the score was not adapted into clinical routine. Importantly, “Cushing symptoms”—and even the combination of these symptoms—occur quite frequently in the general population (see Table 1).

**Table 1:** Clinical symptoms in Cushing's syndrome [*Prevalence in Cushing's syndrome adapted from Valassi et al. *(**19**), Sharma et al*. *(**20**), Feelders et al*. *(**21**), and Newell-Price et al*. *(**2**)*] and in the general population.

**Symptom**	Prevalence in Cushing's syndrome*	**Prevalence in the general population**
**OBJECTIVELY ASSESSABLE**		
Recent weight gain	70–95%	Up to 50% (22)
Overweight	21–48%	50%
Obesity	32–41%	20%
Hypertension (blood pressure > 140/90 mm mercury)	58–85%	Up to 44% (23)
Abnormal glucose tolerance	21–64%	7.4–16.4% (24)
Diabetes mellitus	20–47%	Age-dependent; 2–22% (25)
Osteopenia	60–80%	Dependent on age, sex; up to 30% in postmenopausal women (26), quite seldom in young men and women
Osteoporosis	31–50%	
Osteoporotic fractures	Asymptomatic fractures up to 80%	
Proximal muscle weakness, myopathy	60–82%	Unclear, dependent on medication, neurological diseases, and alcohol abuse
Dyslipidemia	38–71%	22% (27)
Purple striae	78% (children)	In healthy men: 11% (28)
Hypercoagulopathy (hemostatic abnormalities)	54%	Variable dependent on diseases
Atherosclerotic changes	27–31%	25% (29)
Round face	81–90%	Unclear
Plethora	70–90%	Unclear
Buffalo hump	50%	Unclear
Acne	59% (children)	12% in women, 3% in men (30)
Hirsutism	56–75%	Unclear, dependent on other diseases and ethnic, up to 83% in PCOS (31)
Stroke	6%	Age- and sex-dependent, 32–99/100.000/year (32)
Myocardial infarction	2%	About 5% lifetime-risk (33)
Major depression	50–81%	10–20% (34)
Lethargy, depression, labile mood	36%, maybe up to 80% (35)	Up to 50% in elderly people (36)
Cognitive changes	Prevalence unclear, but cognitive impairments are frequent even over the long-term (37)	Unclear
Hypocampal atrophy	27% (38)	High in patients with dementia or Alzheimer's disease, unclear in the younger population
Thrombosis	Incidence: 2.5–14.6/1,000 persons/year	Incidence: 1/1,000; higher in older people (39)
Lung embolism	4%	0,5/1.000 (40)
Nephrolithiasis	20–50%	1–20% (dependent on country) (41)
Thin skin	37%	Unclear
Lack of vitamin D	Unclear, but at least as frequent as in the general population	40–100% (42)
Edema	Unclear	Dependent on disease
Decreased growth in children	70–80%	3%
Asymptomatic urinary tract infections	Unclear	Very common (43)
Sleeping disorder, fatigue	60%	Up to 25% (44)
Easy bruising	35–65%	Unclear
Decreased libido	24–80%	29% (women) (45)
Poor wound healing	Unclear	Unclear
Menstrual changes (women)	70–80%	Dependent on illness
Hair loss	31%	Up to 65% (46)

More recently, Leon-Justel et al. developed a risk scoring system based on a prospective multicenter screening of 353 patients. Thirteen university hospitals in Spain took part in the study. All these patients consulted an endocrinologist due to different medical problems but not with the suspicion of CS. Patients were included in the screening when they had at least two of the following symptoms: obesity, a poorly controlled blood pressure, an uncontrolled diabetes, virilization syndrome, or osteoporosis. For screening purposes, the late-night salivary cortisol and the low-dose dexamethasone suppression test (LDDST) were used. Finally, 26 patients with CS were identified among this cohort (7.4%). Diagnosis was confirmed by a full biochemical screening and follow-up. The clinical features of patients with CS and patients without it were compared. In a univariate analysis, three clinical signs were significantly associated with CS: muscular atrophy, osteoporosis, and dorsocervical fat pad. Two clinical signs—obesity and type 2 diabetes—were more common in patients without a CS than in patients with CS. Different statistical models were studied, leading to a final score with just three clinical signs and one laboratory parameter: osteoporosis, dorsocervical fat pad, muscular atrophy, and late-night salivary cortisol levels (47). Muscular atrophy is higher scored than the other two clinical parameters, while salivary cortisol is divided into low, medium, and high with different scores. The maximum is a score of 12 points. The final form was as follows:

*Risk for CS* = *2* × *presence of osteoporosis (yes/no)* + *2* × *dorsocervical fat pad (yes/no)* + *3* × *muscular atrophy (yes/no)* + *late-night salivary cortisol (low* = *0, medium* = *4, high* = *5). Using 4 points as a cutoff, sensitivity of this score is 96% and specificity is 83%* (47).

This score is quite promising, as false-negative results are rare. However, specificity is not sufficiently high, so that further tests have to be conducted to confirm or exclude the diagnosis. Additionally, it is not described if muscular atrophy and osteoporosis were assessed objectively (for example, by a densitometry of a hand-grip test for muscle strength) or just by the medical history.

### Automated Face Recognition

Face classification was primarily used to identify patients with acromegaly, for example, in a study by Learned-Miller et al. in 2006 (48). In a more recent study, accuracy rates up to 72% were achieved. Especially in patients with less-severe diseases, software classification outperformed medical experts significantly (49).

Face classification was also already used to distinguish between patients with CS and patients with metabolic syndrome, but only exploratively in a small cohort (50). Automatic face classification can detect CS in 85% of patients correctly and excludes the disease in 95% correctly. However, further studies with BMI-matched controls are needed to evaluate the use of automatic face recognition in CS (51). In a recent follow-up study, we enrolled 82 patients (22 male, 60 female) and 98 control subjects (32 male, 66 female) rigidly matched by age, gender, and body mass index. The control group consisted of patients with initially suspected, but biochemically excluded CS. The images were analyzed using specialized computer vision and classification software. The overall correct classification rates were 46 and 81% for male patients and controls, and 57 and 65% for female patients and controls, respectively. This moderate diagnostic accuracy is probably related to the clinical characteristics of the control group (“rule-out CS patients”) who have shown some signs of CS, whereas discrimination form a normal control group would have been more efficient (52).

### Extended Screening Approach for CS

Another screening approach to shorten time to diagnosis has been based on the assumption that the disease may be prevalent in an early form in certain at-risk population, such as the metabolic syndrome. It was based on the rationale that the prevalence of CS is expected to be high and screening those patient cohorts might detect cases in an oligosymptomatic state allowing before evolution of full-blown phenotype. Typical patient cohorts screened were patients with type 2 diabetes, uncontrolled hypertension, osteoporosis, or advanced metabolic syndrome. This concept, initially promoted by data from small studies with selection bias, has been called into question since the incidence of CS was quite low in extended studies.

In unselected patients with diabetes mellitus type 2, CS is very rare. In a study by Reimondo et al., a group of 100 patients with newly diagnosed diabetes mellitus was screened for CS. One patient had biochemical evidence of hypercortisolism but without any clinical features (53). In a study by Mullan et al., 79 controls and 201 patients with diabetes were screened for CS. None of these patients was diagnosed with CS (54). In a large multicenter study in Italy, 813 patients with diabetes were screened. 0.6% (6 persons) had a hypercortisolism but only four of them were successfully cured from CS by surgery (55). In altogether seven studies, a prevalence between 0 and 3% was reported (56).

In two studies, the prevalence of CS among patients with hypertension has been analyzed: a study from the US identified CS in 0.5% of 4,429 patients (57). In a study from Japan, over 1,000 patients were screened and about 1% were diagnosed with CS (58).

Surprisingly, 10% of patients with obesity were diagnosed with CS in one study (59), but these results are probably not reliable as standard screening tests can be falsely positive in very obese patients (60). The results of screening studies among obese patients are very controversial, most likely due to different methodological flaws in these studies according to a systematic literature review by Tabarin and Perez (56). All in all, screening for CS in this group does not seem to be efficient.

However, there are two groups, in which screening for CS is actually reasonable: young patients with osteoporosis and patients with adrenal incidentaloma.

CS is quite common in patients with osteoporosis and vertebral fractures: about 5% of these patients have CS, mostly caused by an adrenal adenoma (61). In another study with 219 patients, 11% of patients with vertebral fractures and a *T* score lower than 2.5 suffered from CS (62). Patients with an adrenal incidentaloma are another group, where standardized screening for CS is endorsed by current guidelines, as on average 5% of the patients have an autonomous cortisol secretion (63), in some studies even up to 10% (64). Screening studies for patients with depression are not available.

In conclusion, widespread screening appears to be neither efficient nor indicated. The prevalence of the metabolic syndrome among US-Americans is 22%. This is far more often than CS, even if the prevalence of the disease might be underestimated (65). Therefore, testing every patient with metabolic syndrome is uneconomical and inefficient and would be associated with an unreasonable number of false-positive results. Finally, physiological forms of hypercortisolism are quite common in a variety of conditions and diseases. Most of them are far more common than CS (see Table 2). However, biochemical testing may be indistinguishable from true CS. This reinforces the paradigm that the confirmed diagnosis of Cushing's syndromes requires both a clinical phenotype and biochemical confirmation.

**Table 2:** States of physiologic hypercortisolism (66, 67).

**Causes**	**Prevalence of the condition in target populations**
•Pregnancy	**–**
•Depression	8.6% (34) in general population
•Alcohol dependence	4.7% (68) in general population
•Glucocorticoid resistance	Unclear, but probably quite seldom
•Obesity	20% (US) in general population, dependent on country (22)
•Diabetes mellitus	6.4% (estimated) in general population (25)
•Physical stress	Unclear
•Malnutrition	Up to 40% in hospitalized patients (69)
•CBG excess	Unclear

## Guidelines for Screening

There are only few guidelines regarding CS. The US Endocrine Society Practice guideline recommends a restricted screening approach for CS in the following instances (66):

- Patients with many clinical signs and symptoms that are typical for CS should be further tested.
- Patients with symptoms that are unusual for their age should be screened.
- Exceptional cases are children with diminished growth and increasing weight. CS should be considered early to avoid short stature in those children.
- Additionally, patients with an adrenal incidentaloma that could be an adenoma should be screened.
- In all other patient groups, regular screening is not recommended.

## Clinical Signs and Symptoms

Many symptoms can go along with CS. Some of them are very common but not very specific, while others are quite specific but not common. Upfront, it is essential to realize that neither the appearance of a single symptom can by itself prove the disease nor the lack of a particular sign can exclude it. In the clinical reasoning process, the entire clinical appearance should be taken into account. The duration and extent of the disease influence the clinical symptomatology (2). Also, sex and age affect clinical presentation (70). That explains the different appearance of patients with the same disease. A few symptoms can be helpful to discriminate patients with metabolic syndrome from CS. Most of them are related to increased protein breakdown due to the underlying catabolic state in hypercortisolism: wide purple striae, plethora, proximal muscle weakness, easy bruising (without a cause), and osteoporosis (without any other reason) (21, 67). In particular, patients with severe CS suffer from proximal muscle weakness and purple striae.

## Clinical Reasoning Should be Simplified According to the Following Principles

There are symptoms and signs that are quite typical for patients with CS as they seldom occur in healthy patients or patients with different diseases:

- Careful inspection of the skin: whereas no patient with CS has all symptoms, it is extremely likely that there will be at least some of the following skin changes (purple striae, ecchymosis, thin fragile skin, rubeosis, hirsutism, acne, impaired wound healing) (2, 21, 67, 71). Purple striae: The prevalence of striae among patients with CS is unclear, but lower than 50%. Its appearance, however, is quite specific for this disease. Cushing striae are livid and at least 1 cm wide. White, reddish, and smaller striae can occur in patients with CS but are far more often caused by obesity, pregnancy, or a fast growth in childhood (72).
- Careful watch-out for signs and symptoms of protein catabolism: Proximal muscle weakness (60–82%) affects usually the muscles of the thighs; patients have problems with stair climbing or squats. As patients often do not discriminate between problems climbing stairs caused by dyspnea or muscle weakness, physicians have to ask these aspects quite specifically when taking the history (73). Other catabolic signs include atrophic thin skin and osteoporosis (see below).
- Plethora (70–90%) (21, 74): Plethora can also be caused by mitral stenosis and different dermatological diseases (such as rosacea or lupus, which generally produce distinct erythema). Patients should be asked whether the color of their skin has changed. Photos of the patient taken a few years ago can help to determine the beginning of the hypercortisolism.
- Atypical fat distribution: in addition to intra-abdominal fat accumulation, the patients have increased fat pads in the buccal area (“moon face”), the neck, the fossa supraclavicularis, and the upper back (“buffalo hump”).
- Atypical symptoms for a specific age can be a hint for Cushing's disease: this includes osteoporotic fractures in pre-menopausal women and men under 50 years (prevalence up to 80% in fluoride CS) (61, 75, 76) and early onset arterial hypertension in patients below 40 years of age, after exclusion of other conditions like hyperaldosteronism or renal arterial stenosis (58).

Some symptoms can be caused by hypercortisolism, but patients might not be aware of this and do not mention them spontaneously:

- Change of taste perception and sense of smell.
- Cognitive changes, including forgetfulness (38).

## Differential Diagnosis of Cushing Phenotypes

All the clinical signs and symptoms mentioned above allow a few differential diagnoses—most of them are much more common than CS and should be considered and possibly excluded (see Table 3). Common differential diagnoses are a PCO syndrome in young women, the metabolic syndrome, and depression. It can be difficult to distinguish between PCO syndrome and CS. We recommend that young women should undergo a gynecological examination. Also, androstenedione, testosterone, and insulin should be measured. In doubt or in biochemical unclear situations, a short-term follow-up (exemplarily after 3 months) can be helpful to differentiate the diagnoses. Depression and diabetes mellitus can also cause mild hypercortisolism. In these cases, a sufficient therapy (for example, with an anti-depressive medication) is helpful to distinguish between diagnoses. Naturally, a careful medication history should be taken to avoid overlooking an exogenous CS.

**Table 3:** Differential diagnosis of Cushing's signs and symptoms.

**Symptom/Sign**	**Possible differential diagnosis (selection)**	
Increased visceral fat tissue, weight gain	• Eating disorder • Pregnancy • Alcohol abuse • PCO syndrome • Seldom: genetic disorders	• Hypothyroidism • Edema • Medication • Insulinoma
Hypertension	• Essential hypertension • Pheochromocytoma-Paragangioma syndrome • Hyperthyroidism • Medication	• Primary aldosteronism • Renal artery stenosis • Sleep apnea • Renal hypertension • Acromegaly
Impaired glucose tolerance, diabetes	• Diabetes mellitus type 1 and 2 • Other endocrinological disorders (hyperthyroidism, acromegaly)	• Gestational diabetes • Chronic pancreatitis • Medication • PCO syndrome
Proximal muscle weakness, myopathy	• Age (sarcopenia) • Alcohol abuse	Neurological disorders
Osteoporosis, osteopenia, fractures	• Postmenopausal osteoporosis • Medication • Primary hyperparathyroidism • Hyperthyroidism	• Senile osteoporosis • Mastozytose • Idiopathic • Malabsorption • Hypogonadism
Dyslipidemia	• Lifestyle • Diabetes mellitus • Liver and renal diseases	• Familial forms • Medication • Alcohol abuse • Obesity
Livid striae	• Obesity • Pregnancy	• Medication • Idiopathic
Round face	Obesity	
Plethora	• Rosacea • Lupus	Mitral stenosis
Buffalo neck	• Madelung Syndrome	Obesity
Acne	• PCO syndrome • Medication	Idiopathic
Hirsutism	• PCO syndrome • Ovarian tumors • Acromegaly • Congenital adrenal hyperplasia	• Medication • Menopause • Idiopathic • Obesity • Diabetes
Lethargy, depression, labile mood	Depression	Other psychiatric disorders
Cognitive changes	• Depression • Age	Dementia
Thrombosis, lung embolism	• Provoked (e.g., immobilization) • Genetic disorders	• Oral contraception • Nicotine abuse
Nephrolithiasis	Medication	Nutrition
Thin skin	• Age • Diabetes mellitus	Medication
Edema	• Heart insufficiency • Lymphedema	Renal insufficiency, other renal disorders
Sleeping disorder	• Depression • Sleep apnea • Alcohol abuse	• Physical andpsychological stress • Hyperthyroidism
Decreased libido	• Depression • Relationship problems • Medication • Adrenal insufficiency	• Physiological stress • Alcohol consume • Hypothyroidism
Recurrent infections	Other immune-deficiencies	HIV infection
Poor wound healing	• Malnutrition • PAVK • Medication • Cancer	• Age • Diabetes mellitus • Nicotine abuse • Anemia
Menstrual changes	• PCO syndrome • Hypo- and hyperthyroidism • AGS • Adrenal insufficiency • Prolactionoma	• Pregnancy • Menopause • Medication • Weight loss • Anorexia
Hair loss	• Androgenic alopecia • Adrenal insufficiency • Lupus • Malnutrition	• Divers dermatologicdisorders • Hyperparathyroidism • Medication

## General Lab Results

A broad laboratory investigation should be conducted as a few changes in laboratory parameters can occur in patients with CS—frequencies unfortunately unclear—and can be a first hint for a hypercortisolism (see Table 4). Hypokalemia occurs in severe hypercortisolism (up to 100% in patients with ectopic CS, 5–10% in Cushing's disease) (77).

**Table 4:** Laboratory changes in patients with Cushing's syndrome.

**Lab parameter**	**Differential diagnoses (selection)**
Hypokalemia (77)	Primary hyperaldosteronism, medication, renal diseases
Low testosterone (78)	Medication, hypogonadism
Eosinopenia (79)	Infections, stress, medication
Hypercalciuria (80)	Hyperparathyroidism, vitamin D intoxication, bone metastasis, sarcoidosis, idiopathic, renal resorption disorders
Leucocytosis, lymphopenia (81)	(Chronic) Infections, HIV, leukemia
Erythrocytosis (82)	Hypoxemia, renal, paraneoplastic

## Biochemical Screening for CS

The US Endocrine Society Practice guideline (66) recommends three tests for biochemical screening: the 1-mg LDDST, the 24-h urine cortisol (UFC) determination, and the late-night salivary cortisol measurement. All other screening methods, such as the 2-mg dexamethasone suppression test, the midnight serum cortisol, or the desmopressin stimulation test, either have a lower sensitivity or are challenging to perform correctly. Therefore, these methods are not recommended for initial screening purposes. Also, the measurement of basal morning serum cortisol or basal ACTH is not diagnostically conclusive because of the diurnal rhythm with physiologically high morning levels. Only in severe cases (for example in ectopic CS) are basal cortisol levels elevated on a regular basis.

## Sensitivity and Specificity of Biochemical Screening Tests

The abovementioned three screening tests all have a comparable accuracy (93). At least two of the screening tests should be performed. When two of them show an abnormal test result AND the patient has a high pre-test probability by clinical presentation, the diagnosis can be confirmed. The sensitivity and the specificity of individual tests is highly depending on factors such as the cutoffs and the assays used. Generally, higher sensitivity can be reached by lower cutoffs, but decreasing specificity—and the other way around. Sensitivity and specificity are almost equal in all three screening tests (shown in Table 5). However, not every test is as useful as the others in all patients (see Tables 6, 7).

**Table 5:** Sensitivity and specificity of first-line screening [adapted from Nieman et al. (66) and Reimondo et al. (83)].

**Tests**	**Sensitivity (%)**	**Specificity (%)**	**Cutoffs**
Low-dose dexamethasone suppression test	80–95	80–95	Depended on assay
Urine free cortisol	45–71	Up to 100	
Late-night salivary cortisol	92–100	85–100	

**Table 6:** Biochemical tests: causes for false-positive and false-negative results (20, 84–92).

**Screening test**	**False positive**	**False negative**
Low-dose dexamethasone suppression test	Oral estrogens, increased CBG, medication, rapid metabolizer, lack of resorption of dexamethasone	Liver or renal failure
Urine free cortisol	Depended on the assay, volume over 5 L, medications	Dependent on the assay, GFR low, improper collection
Salivary cortisol	Age, hypertension, diabetes, dependent on the assay, depression, shift workers, smokers, stress, maybe blood	-

**Table 7:** Which screening method should be performed in which patient? [adapted from Nieman et al. (66)].

**Patient group**	**Recommended methods**	**False-positive results**	**False-negative results**
Patients with renal impairment	LDDST, salivary cortisol	-	UFC
Women under contraception	UFC	LDDST	-
Smokers	UFC, LDDST	Salivary cortisol	-
Mild or cyclic Cushing	Repeated measurements of UFC and/or salivary	-	LDDST
Adrenal Cushing	Salivary cortisol, LDDST	-	UFC
Pregnancy	UFC	-	LDDST
Patients with epilepsy	UFC, salivary cortisol	LDDST	-

## False-Positive and False-Negative Biochemical Test Results

### Low-Dose Dexamethasone Suppression Test

The LDDST can be falsely positive in women who take oral contraception. Up to 50% of these women will have a positive test result (3). The oral contraception causes an increased CBG, which influences the test results (84). In these women, the cortisol measurement in the urine is more helpful as CBG levels do not affect it. Otherwise, oral contraception should be paused at least 1 week but ideally for 3 months. Mitotane also increases CBG levels and may lead to incorrect test results. Other medications can influence the test results by induction or inhibition of CYP 3A4. Aprepitant, itraconazole, ritonavir, and diltiazem, for example, slow down dexamethasone metabolism (66). Phenobarbital, phenytoin, carbamazepine, rifampicin, and pioglitazone, however, will increase dexamethasone metabolism (66). Additionally, individual and variable absorption and metabolism of dexamethasone has become recently a focus and will have an impact on test results. A measurement of dexamethasone levels in spot urine or plasma can be helpful to evaluate the validity of adequate resorption and metabolism (94). Finally, liver or renal insufficiency may lead to reduced dexamethasone clearance, which could cause a false-negative test result.

### Urine Free Cortisol (UFC)

The UFC has been used for over 50 years to diagnose CS (85). Unlike plasma cortisol, CBG levels do not affect the results as the free hormone is measured and not the CBG bound form. Therefore, UFC is the preferred test for women taking oral contraceptives. Nevertheless, different conditions can also lead to false-positive and -negative test results. A fluid intake of over 5 L per day increases cortisol levels (95), while an incomplete collection of the urine may cause false-negative test results. Also, the selection of urine is not useful in patients with chronic renal insufficiency, as UFC levels decrease with a decreasing creatinine clearance (86). Similar to the LDDST, medication can influence the test results. For example, carbamazepine, fenofibrate, and synthetic glucocorticoids increase UFC results. Because of day-to-day fluctuations, current guidelines suggest collection of at least two 24-h urines.

Depending on calibration characteristics or cross-reactivity of first antibody with other steroids, commercial assays can both lead to false-positive and false-negative test results (83–85, 87).

### Late-Night Salivary Cortisol

The sensitivity and specificity of salivary cortisol are described in the literature as quite high, but they are very much influenced by the methodology. Specificity varies between 85 and 100% depending on assay characteristics and technique. Hypertension and diabetes can cause elevated cortisol levels in up to 40% (88) as well as depression (89). Shift working may also be a reason for an impaired circadian rhythm. Smoking leads to elevated cortisol levels in saliva and should be avoided on the day of collection (90). Bleeding or oral infections can also influence the test results. Therefore, teeth brushing (which might lead to blood leaks) should be avoided before sampling the salivary. Because of a higher rate of test variability, collection of two late-night salivary cortisol samples is recommended.

### Assay Problems

To make matters worse, the test results depend on the specific assays used to measure hormone concentrations. Basal cortisol (96), ACTH (97), and urine cortisol (98) levels diverge by up to 50% depending on the assays employed, heavily influencing classification of patients. Also, salivary cortisol levels are not comparable between assays from different manufacturers (99). Especially in women on oral contraception, different assays can result in highly discrepant results (100). Cortisol levels from different laboratories are only comparable if the same assay is used. Accordingly, recommended cutoffs and decision limits need to be defined in an assay-specific manner. Exemplarily, cortisol can be measured using immunoassays, gas chromatography mass spectrometry (GC-MS), or liquid chromatography mass spectrometry (LC-MS). Cortisol levels as measured by the latter two methods commonly are reported to be lower (101) than those obtained by immunoassays. One reason is that immunoassays, particularly direct ones, can suffer from cross-reactions and thus have less specificity than the other two methods (101). The resulting over- or underestimation of cortisol concentrations can be a serious problem (96). Nevertheless, in clinical routine, immunoassays still are the most frequently used method (101). The same problems apply to the use of salivary cortisol, where concentrations measured by LC-MS and immunoassays exhibit poor agreement (92, 101).

## Summary of the First-Line Screening

**Figure 1:**
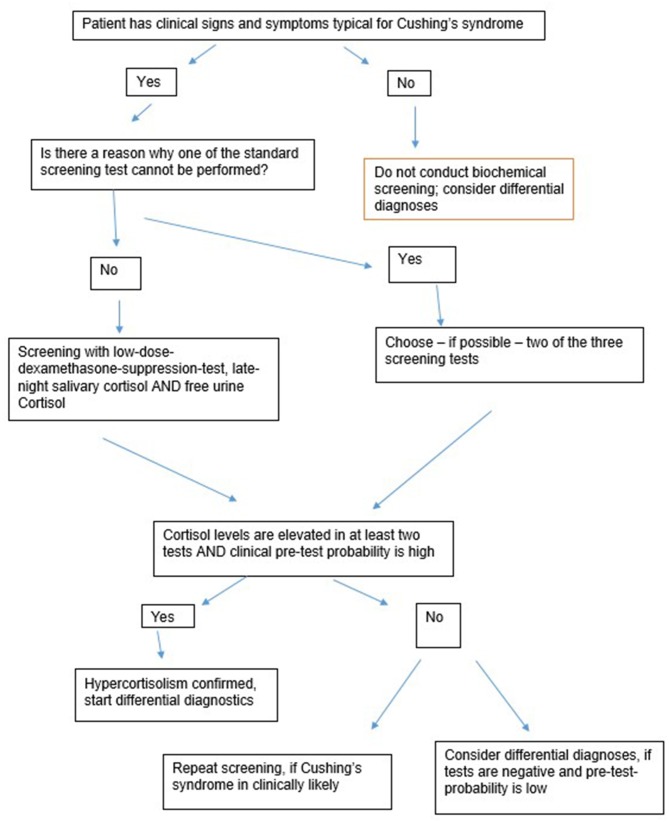
Overview diagnostic steps.

Figure 1 gives an overview about the first-line-screening-steps. The following rules are essential regarding the first-line-screening:

1. Biochemical screening should only be conducted when the patient has clinical features suggesting CS.
2. At least two tests should be conducted.
3. False-negative and false-positive results are likely. A positive test result is therefore not a proof for a CS.
4. When in doubt, biochemical screening should be repeated.

## Exceptional Cases: Diagnostics in Mild and Episodic CS

Severe hypercortisolism can be relatively easily detected using the tests mentioned above. Mild or cyclic CS, however, requires a different procedure. Recent studies suggest that mild or episodic hypercortisolism is more frequent than assumed (102). In these cases, the clinical signs or symptoms of the patient are even more critical. If clinically a CS is likely, a single negative test should not lead to the exclusion of an endogenous hypercortisolism. Short-term follow-ups are essential to confirm or exclude the diagnosis. Diagnosing patients with just a few—or maybe even one—“Cushing symptoms” can be challenging as well. It is important to consider CS as a possible differential diagnosis in young patients with osteoporosis, hypertension, or even mental disorders that cannot be explained by anything else.

Just a few studies focused on cyclic CS so far. Generally, repeating the sampling of urine or salivary is recommended while the LDDST is less useful (102, 103). We recommend to carefully take a history in these patients to investigate whether they can identify a time pattern in their symptoms. Additionally, as it can be easily conducted at home, these patients should collect salivary samples or urine over a longer time period. Also, measuring cortisol in the hair has been advocated to detect cyclic CS (104). This assay is currently not commercially available.

## New Approaches: Plasma Steroid Metabolome Profiling

Using LC-MS, we recently explored the diagnostic accuracy of steroid profiling of basal plasma samples from patients with CS. A panel of 15 plasma steroids were applied to 222 patient samples tested for CS. Disease was excluded in 138 and confirmed in 51 patients with pituitary CS, 12 with ectopic adrenocorticotropin secretion, and 21 with adrenal CS. We identified distinct steroid profiles among patients with CS that separated patients from healthy controls. Moreover, classification into the three Cushing subtypes was achieved with reasonable accuracy. With the use of 10 selected steroids, subjects with and without different CS subtypes could be discriminated nearly as good as with the use of a combination of the three conventional screening tests and plasma ACTH (misclassification of 9.5% of subjects with steroid profiling vs. 5.8% with conventional testing) (105). In another study, we demonstrated that the 15-plasma-steroid panel was able to discriminate 35 patients with subclinical CS from 21 with overt clinical CS and normal controls. Patients with subclinical CS had lower plasma concentrations of dehydroepiandrosterone and dehydroepiandrosterone-sulfate than normal subjects, but higher levels of 11-deoxycortisol and 11-deoxycorticosterone. The steroid combination provided superior diagnostic performance compared to each of the other routine biochemical tests (106). These data are in line with recent data obtained in patients with primary aldosteronism (107) and could pave the way to simplified biochemical confirmation of steroid excess syndromes if confirmed by other studies.

## Conclusion and Outlook

Diagnosing CS is not straightforward: it remains challenging to preselect patients with an increased likelihood of CS and to select the best biochemical screening test for a given patient. Undoubtedly, current clinical and biochemical screening approaches are far from ideal, and simplified procedures are required from both a patient's and a physician's perspective. The clinical score by Leon-Justel could be a good starting point but it should be validated in other patient cohorts. Other laboratory tests like plasma steroid profiling are promising approaches to reduce the number of diagnostic tests (105).

To conclude, we suggest a rational clinical score for patients with CS: based on the scores by Leon-Justel and Nugent and the results from different screening studies, the following clinical signs should be included in a clinical score: osteoporosis, muscular atrophy [objectively assessed by a combination of the chair rising test, hand-grip measurement, and the bioelectrical impedance measurement (8)], livid striae, plethora, ecchymoses, and dorsocervical fat pat. Such a clinical score should be validated comparing a cohort of patients with CS and patients with an initial clinical suspicion for hypercortisolism in which CS was excluded.

## Author Contributions

LB and MR have written the first draft of the manuscript. AR, AO-K, JF, GR, MB, and FB substantially contributed to the revision of the manuscript. All authors read and approved the final version for submission.

### Conflict of Interest

The authors declare that the research was conducted in the absence of any commercial or financial relationships that could be construed as a potential conflict of interest.

## References

[1] Steffensen C Bak AM Rubeck KZ Jorgensen JO. Epidemiology of Cushing's syndrome. Neuroendocrinology. (2010) 92 (Suppl. 1):1–5. 10.1159/000314297 20829610

[2] Newell-Price J Bertagna X Grossman AB Nieman LK. Cushing's syndrome. Lancet. (2006) 367:1605–17. 10.1016/S0140-6736(06)68699-6 16698415

[3] Debono M Newell-Price JD. Cushing's syndrome: where and how to find it. Front Horm Res. (2016) 46:15–27. 10.1159/000443861 27211887

[4] Kreitschmann-Andermahr I Psaras T Tsiogka M Starz D Kleist B Siegel S From first symptoms to final diagnosis of Cushing's disease: experiences of 176 patients. Eur J Endocrinol. (2015) 172:X1 10.1530/EJE-14-0766 25976214

[5] Javanmard P Duan D Geer EB. Mortality in patients with endogenous Cushing's syndrome. Endocrinol Metab Clin North Am. (2018) 47:313–33. 10.1016/j.ecl.2018.02.005 29754634

[6] Valassi E Tabarin A Brue T Feelders RA Reincke M Netea-Maier R High mortality within 90 days of diagnosis in patients with Cushing's syndrome - Results from the ERCUSYN registry. Eur J Endocrinol. (2019) 181:461–72. 10.1530/EJE-19-0464 31480014

[7] Berr CM Di Dalmazi G Osswald A Ritzel K Bidlingmaier M Geyer LL. Time to recovery of adrenal function after curative surgery for Cushing's syndrome depends on etiology. J Clin Endocrinol Metab. (2015) 100:1300–8. 10.1210/jc.2014-3632 25546155

[8] Berr CM Stieg MR Deutschbein T Quinkler M Schmidmaier R Osswald A. Persistence of myopathy in Cushing's syndrome: evaluation of the German Cushing's Registry. Eur J Endocrinol. (2017) 176:737–46. 10.1530/EJE-16-0689 28325824

[9] Osswald A Deutschbein T Berr CM Plomer E Mickisch A Ritzel K. Surviving ectopic Cushing's syndrome: quality of life, cardiovascular and metabolic outcomes in comparison to Cushing's disease during long-term follow-up. Eur J Endocrinol. (2018) 179:109–16. 10.1530/EJE-18-0212 29875286

[10] Rubinstein G Osswald A Hoster E Losa M Elenkova A Zacharieva S. Time to diagnosis in Cushing's syndrome: a meta-analysis based on 5367 patients. J Clin Endocrinol Metab. (2019) dgz136. 10.1210/clinem/dgz136 31665382

[11] Elstein AS Shulman LS Sprafka SA Medical Problem Solving an Analysis of Clinical Reasoning. Cambridge: Harvard University Press (1978). 10.4159/harvard.9780674189089

[12] Braun LT Zottmann JM Adolf C Lottspeich C Then C Wirth S. Representation scaffolds improve diagnostic efficiency in medical students. Med Educ. (2017) 51:1118–26. 10.1111/medu.13355 28585351

[13] Graber ML Carlson B. Diagnostic error: the hidden epidemic. Physician Exec. (2011) 37:12–4, 6, 8–9. 22195411

[14] Zwaan L de Bruijne M Wagner C Thijs A Smits M van der Wal G. Patient record review of the incidence, consequences, and causes of diagnostic adverse events. Arch Intern Med. (2010) 170:1015–21. 10.1001/archinternmed.2010.146 20585065

[15] Graber ML Franklin N Gordon R. Diagnostic error in internal medicine. Arch Intern Med. (2005) 165:1493–9. 10.1001/archinte.165.13.1493 16009864

[16] Schmidt HG Rikers RM. How expertise develops in medicine: knowledge encapsulation and illness script formation. Med Educ. (2007) 41:1133–9. 10.1111/j.1365-2923.2007.02915.x 18004989

[17] Custers EJ. Thirty years of illness scripts: theoretical origins and practical applications. Med Teach. (2015) 37:457–62. 10.3109/0142159X.2014.956052 25180878

[18] Nugent CA Warner HR Dunn JT Tyler FH. Probability theory in the diagnosis of Cushing's syndrome. J Clin Endocrinol Metab. (1964) 24:621–7. 10.1210/jcem-24-7-621 14212081

[19] Valassi E Santos A Yaneva M Toth M Strasburger CJ Chanson P. The European Registry on Cushing's syndrome: 2-year experience. Baseline demographic and clinical characteristics. Eur J Endocrinol. (2011) 165:383–92. 10.1530/EJE-11-0272 21715416

[20] Sharma ST Nieman LK Feelders RA. Cushing's syndrome: epidemiology and developments in disease management. Clin Epidemiol. (2015) 7:281–93. 10.2147/CLEP.S44336 25945066 PMC4407747

[21] Feelders RA Pulgar SJ Kempel A Pereira AM. The burden of Cushing's disease: clinical and health-related quality of life aspects. Eur J Endocrinol. (2012) 167:311–26. 10.1530/EJE-11-1095 22728347

[22] Mokdad AH Ford ES Bowman BA Dietz WH Vinicor F Bales VS. Prevalence of obesity, diabetes, and obesity-related health risk factors, 2001. JAMA. (2003) 289:76–9. 10.1001/jama.289.1.76 12503980

[23] Wolf-Maier K Cooper RS Banegas JR Giampaoli S Hense HW Joffres M. Hypertension prevalence and blood pressure levels in 6 European countries, Canada, and the United States. JAMA. (2003) 289:2363–9. 10.1001/jama.289.18.2363 12746359

[24] Dunstan DW Zimmet PZ Welborn TA De Courten MP Cameron AJ Sicree RA. The rising prevalence of diabetes and impaired glucose tolerance: the Australian Diabetes, Obesity and Lifestyle Study. Diabetes Care. (2002) 25:829–34. 10.2337/diacare.25.5.829 11978676

[25] Shaw JE Sicree RA Zimmet PZ. Global estimates of the prevalence of diabetes for 2010 and 2030. Diab Res Clin Pract. (2010) 87:4–14. 10.1016/j.diabres.2009.10.007 19896746

[26] Reginster J-Y Burlet N. Osteoporosis: a still increasing prevalence. Bone. (2006) 38:4–9. 10.1016/j.bone.2005.11.024 16455317

[27] Kit BK Kuklina E Carroll MD Ostchega Y Freedman DS Ogden CL. Prevalence of and trends in dyslipidemia and blood pressure among US children and adolescents, 1999–2012. JAMA Pediatrics. (2015) 169:272–9. 10.1001/jamapediatrics.2014.3216 25599372 PMC7423159

[28] Elton RF Pinkus H. Striae in normal men. Arch Dermatol. (1966) 94:33–4. 10.1001/archderm.1966.01600250039005 5938220

[29] Prati P Vanuzzo D Casaroli M Di Chiara A De Biasi F Feruglio GA. Prevalence and determinants of carotid atherosclerosis in a general population. Stroke. (1992) 23:1705–11. 10.1161/01.STR.23.12.1705 1448818

[30] Goulden V Stables GI Cunliffe WJ. Prevalence of facial acne in adults. J Am Acad Dermatol. (1999) 41:577–80. 10.1016/S0190-9622(99)70300-2 10495379

[31] Falsetti L Gambera A Andrico S Sartori E. Acne and hirsutism in polycystic ovary syndrome: clinical, endocrine–metabolic and ultrasonographic differences. Gynecol Endocrinol. (2002) 16:275–84. 10.1080/gye.16.4.275.284 12396556

[32] Feigin VL Lawes CM Bennett DA Anderson CS. Stroke epidemiology: a review of population-based studies of incidence, prevalence, and case-fatality in the late 20th century. Lancet Neurol. (2003) 2:43–53. 10.1016/S1474-4422(03)00266-7 12849300

[33] Gößwald A Schienkiewitz A Nowossadeck E Busch M Prävalenz von Herzinfarkt und koronarer Herzkrankheit bei Erwachsenen im Alter von 40 bis 79 Jahren in Deutschland. Bundesgesundheitsblatt-Gesundheitsforschung-Gesundheitsschutz. (2013) 56:650–5. 10.1007/s00103-013-1666-9 23703482

[34] Ayuso-Mateos JL Vázquez-Barquero JL Dowrick C Lehtinen V Dalgard OS Casey P. Depressive disorders in Europe: prevalence figures from the ODIN study. Br J Psychiatry. (2001) 179:308–16. 10.1192/bjp.179.4.308 11581110

[35] Kelly WF Kelly MJ Faragher B. A prospective study of psychiatric and psychological aspects of Cushing's syndrome. Clin Endocrinol. (1996) 45:715–20. 10.1046/j.1365-2265.1996.8690878.x 9039337

[36] Djernes JK. Prevalence and predictors of depression in populations of elderly: a review. Acta Psychiatr Scand. (2006) 113:372–87. 10.1111/j.1600-0447.2006.00770.x 16603029

[37] Tiemensma J Kokshoorn NE Biermasz NR Keijser BJ Wassenaar MJ Middelkoop HA. Subtle cognitive impairments in patients with long-term cure of Cushing's disease. J Clin Endocrinol Metab. (2010) 95:2699–714. 10.1210/jc.2009-2032 20371667

[38] Starkman MN Gebarski SS Berent S Schteingart DE. Hippocampal formation volume, memory dysfunction, and cortisol levels in patients with Cushing's syndrome. Biol Psychiatry. (1992) 32:756–65. 10.1016/0006-3223(92)90079-F 1450290

[39] Diehm C Noppeney T Nüllen H Epidemiologie der venösen Thromboembolie. Gefässchirurgie. (2012) 17:275–9. 10.1007/s00772-011-0929-8

[40] Walther A Böttiger B Die akute Lungenarterienembolie. Der Anaesthesist. (2002) 51:427–46. 10.1007/s00101-002-0315-8 12125319

[41] Ramello A Vitale C Marangella M. Epidemiology of nephrolithiasis. J Nephrol. (2001) 13:S45–50. 11132032

[42] Holick MF. Vitamin D deficiency. N Engl J Med. (2007) 357:266–81. 10.1056/NEJMra070553 17634462

[43] Foxman B. Epidemiology of urinary tract infections: incidence, morbidity, and economic costs. Am J Med. (2002) 113:5–13. 10.1016/S0002-9343(02)01054-9 12113866

[44] Ram S Seirawan H Kumar SK Clark GT. Prevalence and impact of sleep disorders and sleep habits in the United States. Sleep Breath. (2010) 14:63–70. 10.1007/s11325-009-0281-3 19629554

[45] De Rose AF Gallo F Bini PM Gattuccio I Chiriaco V Terrone C. Epidemiology of sexual disorders in general medical practice: an Italian survey. Urologia. (2019) 86:79–85. 10.1177/0391560319842955 30983516

[46] Gan DC Sinclair RD. Prevalence of male and female pattern hair loss in Maryborough. J Investig Dermatol Symp Proc. (2005) 10:184–9. 10.1111/j.1087-0024.2005.10102.x 16382660

[47] Leon-Justel A Madrazo-Atutxa A Alvarez-Rios AI Infantes-Fontan R Garcia-Arnes JA Lillo-Munoz JA. A probabilistic model for Cushing's syndrome screening in at-risk populations: a prospective multicenter study. J Clin Endocrinol Metab. (2016) 101:3747–54. 10.1210/jc.2016-1673 27490917

[48] Learned-Miller E Lu Q Paisley A Trainer P Blanz V Dedden K. Detecting acromegaly: screening for disease with a morphable model. Med Image Comput Comput Assist Interv. (2006) 9(Pt 2):495–503. 10.1007/11866763_61 17354809

[49] Schneider HJ Kosilek RP Gunther M Roemmler J Stalla GK Sievers C. A novel approach to the detection of acromegaly: accuracy of diagnosis by automatic face classification. J Clin Endocrinol Metab. (2011) 96:2074–80. 10.1210/jc.2011-0237 21508144

[50] Kosilek RP Schopohl J Grunke M Reincke M Dimopoulou C Stalla GK. Automatic face classification of Cushing's syndrome in women - a novel screening approach. Exp Clin Endocrinol Diabetes. (2013) 121:561–4. 10.1055/s-0033-1349124 23864496

[51] Kosilek RP Frohner R Wurtz RP Berr CM Schopohl J Reincke M. Diagnostic use of facial image analysis software in endocrine and genetic disorders: review, current results and future perspectives. Eur J Endocrinol. (2015) 173:M39–44. 10.1530/EJE-15-0429 26162404

[52] Popp KH Kosilek RP Frohner R Stalla GK Athanasoulia-Kaspar A Berr C. Computer vision technology in the differential diagnosis of Cushing's syndrome. Exp Clin Endocrinol Diabetes. (2019) 127:685–90. 10.1055/a-0887-4233 31158898

[53] Reimondo G Pia A Allasino B Tassone F Bovio S Borretta G. Screening of Cushing's syndrome in adult patients with newly diagnosed diabetes mellitus. Clin Endocrinol. (2007) 67:225–9. 10.1111/j.1365-2265.2007.02865.x 17547690

[54] Mullan K Black N Thiraviaraj A Bell PM Burgess C Hunter SJ. Is there value in routine screening for Cushing's syndrome in patients with diabetes? J Clin Endocrinol Metab. (2010) 95:2262–5. 10.1210/jc.2009-2453 20237165

[55] Terzolo M Reimondo G Chiodini I Castello R Giordano R Ciccarelli E. Screening of Cushing's syndrome in outpatients with type 2 diabetes: results of a prospective multicentric study in Italy. J Clin Endocrinol Metab. (2012) 97:3467–75. 10.1210/jc.2012-1323 22767639

[56] Tabarin A Perez P. Pros and cons of screening for occult Cushing syndrome. Nat Rev Endocrinol. (2011) 7:445. 10.1038/nrendo.2011.51 21423244

[57] Anderson GH Jr Blakeman N Streeten DH. The effect of age on prevalence of secondary forms of hypertension in 4429 consecutively referred patients. J Hypertens. (1994) 12:609–15. 10.1097/00004872-199405000-00015 7930562

[58] Omura M Saito J Yamaguchi K Kakuta Y Nishikawa T. Prospective study on the prevalence of secondary hypertension among hypertensive patients visiting a general outpatient clinic in Japan. Hypertens Res. (2004) 27:193–202. 10.1291/hypres.27.193 15080378

[59] Tiryakioglu O Ugurlu S Yalin S Yirmibescik S Caglar E Yetkin DO. Screening for Cushing's syndrome in obese patients. Clinics. (2010) 65:9–13. 10.1590/S1807-59322010000100003 20126340 PMC2815288

[60] Baid SK Rubino D Sinaii N Ramsey S Frank A Nieman LK. Specificity of screening tests for Cushing's syndrome in an overweight and obese population. J Clin Endocrinol Metab. (2009) 94:3857–64. 10.1210/jc.2008-2766 19602562 PMC2758724

[61] Shimon I. Screening for Cushing's syndrome: is it worthwhile? Pituitary. (2015) 18:201–5. 10.1007/s11102-015-0634-9 25578150

[62] Chiodini I Mascia ML Muscarella S Battista C Minisola S Arosio M. Subclinical hypercortisolism among outpatients referred for osteoporosis. Ann Intern Med. (2007) 147:541–8. 10.7326/0003-4819-147-8-200710160-00006 17938392

[63] Fassnacht M Arlt W Bancos I Dralle H Newell-Price J Sahdev A. Management of adrenal incidentalomas: European Society of Endocrinology Clinical Practice Guideline in collaboration with the European Network for the Study of Adrenal Tumors. Eur J Endocrinol. (2016) 175:G1–g34. 10.1530/EJE-16-0467 27390021

[64] Nieman LK. Approach to the patient with an adrenal incidentaloma. J Clin Endocrinol Metab. (2010) 95:4106–13. 10.1210/jc.2010-0457 20823463 PMC2936073

[65] Ford ES Giles WH Dietz WH. Prevalence of the metabolic syndrome among US adults: findings from the third National Health and Nutrition Examination Survey. JAMA. (2002) 287:356–9. 10.1001/jama.287.3.356 11790215

[66] Nieman LK Biller BM Findling JW Newell-Price J Savage MO Stewart PM. The diagnosis of Cushing's syndrome: an Endocrine Society Clinical Practice Guideline. J Clin Endocrinol Metab. (2008) 93:1526–40. 10.1210/jc.2008-0125 18334580 PMC2386281

[67] Nieman LK. Cushing's syndrome: update on signs, symptoms and biochemical screening. Eur J Endocrinol. (2015) 173:M33–8. 10.1530/EJE-15-0464 26156970 PMC4553096

[68] Hasin DS Stinson FS Ogburn E Grant BF. Prevalence, correlates, disability, and comorbidity of DSM-IV alcohol abuse and dependence in the United States: results from the National Epidemiologic Survey on Alcohol and Related Conditions. Arch Gen Psychiatry. (2007) 64:830–42. 10.1001/archpsyc.64.7.830 17606817

[69] Barker L Gout B Crowe T. Hospital malnutrition: prevalence, identification and impact on patients and the healthcare system. Int J Environ Res Public Health. (2011) 8:514–27. 10.3390/ijerph8020514 21556200 PMC3084475

[70] Giraldi FP Moro M Cavagnini F Endocrinology SGotH-P-AAotISo Gender-related differences in the presentation and course of Cushing's disease. J Clin Endocrinol Metab. (2003) 88:1554–8. 10.1210/jc.2002-021518 12679438

[71] Stratakis CA. Skin manifestations of Cushing's syndrome. Rev Endocr Metab Disord. (2016) 17:283–6. 10.1007/s11154-016-9399-3 27943005 PMC5181654

[72] Elsaie ML Baumann LS Elsaaiee LT. Striae distensae (stretch marks) and different modalities of therapy: an update. Dermatol Surg. (2009) 35:563–73. 10.1111/j.1524-4725.2009.01094.x 19400881

[73] Ross EJ Linch DC. Cushing's syndrome–killing disease: discriminatory value of signs and symptoms aiding early diagnosis. Lancet. (1982) 2:646–9. 10.1016/S0140-6736(82)92749-0 6125785

[74] Afshari A Ardeshirpour Y Lodish MB Gourgari E Sinaii N Keil M. Facial plethora: modern technology for quantifying an ancient clinical sign and its use in Cushing syndrome. J Clin Endocrinol Metab. (2015) 100:3928–33. 10.1210/jc.2015-2497 26301943 PMC4596033

[75] Vestergaard P Lindholm J Jorgensen JO Hagen C Hoeck HC Laurberg P. Increased risk of osteoporotic fractures in patients with Cushing's syndrome. Eur J Endocrinol. (2002) 146:51–6. 10.1530/eje.0.1460051 11751067

[76] Belaya ZE Hans D Rozhinskaya LY Dragunova NV Sasonova NI Solodovnikov AG. The risk factors for fractures and trabecular bone-score value in patients with endogenous Cushing's syndrome. Arch Osteoporos. (2015) 10:44. 10.1007/s11657-015-0244-1 26608406

[77] Stewart PM Walker BR Holder G O'Halloran D Shackleton CH. 11 beta-Hydroxysteroid dehydrogenase activity in Cushing's syndrome: explaining the mineralocorticoid excess state of the ectopic adrenocorticotropin syndrome. J Clin Endocrinol Metab. (1995) 80:3617–20. 10.1210/jc.80.12.3617 8530609

[78] Doerr P Pirke KM. Cortisol-induced suppression of plasma testosterone in normal adult males. J Clin Endocrinol Metab. (1976) 43:622–9. 10.1210/jcem-43-3-622 956348

[79] Sabag N Castrillon M Tchernitchin A. Cortisol-induced migration of eosinophil leukocytes to lymphoid organs. Experientia. (1978) 34:666–7. 10.1007/BF01937022 658264

[80] Faggiano A Pivonello R Melis D Filippella M Di Somma C Petretta M. Nephrolithiasis in Cushing's Disease: prevalence, etiopathogenesis, and modification after disease cure. J Clin Endocrinol Metab. (2003) 88:2076–80. 10.1210/jc.2002-021494 12727957

[81] Hurxthal LM O'sullivan JB. Cushing's syndrome: clinical differential diagnosis and complications. Ann Intern Med. (1959) 51:1–16. 10.7326/0003-4819-51-1-1 13661781

[82] Gursoy A Unal AD Ayturk S Karakus S Izol AN Tutuncu NB. Polycythemia as the first manifestation of Cushing's disease. J Endocrinol Invest. (2006) 29:742–4. 10.1007/BF03344186 17033265

[83] Reimondo G Pia A Bovio S Allasino B Daffara F Paccotti P. Laboratory differentiation of Cushing's syndrome. Clin Chim Acta. (2008) 388:5–14. 10.1016/j.cca.2007.10.036 18053807

[84] Elias PC Martinez EZ Barone BF Mermejo LM Castro M Moreira AC. Late-night salivary cortisol has a better performance than urinary free cortisol in the diagnosis of Cushing's syndrome. J Clin Endocrinol Metab. (2014) 99:2045–51. 10.1210/jc.2013-4262 24628557

[85] Raff H Auchus RJ Findling JW Nieman LK. Urine free cortisol in the diagnosis of Cushing's syndrome: is it worth doing and, if so, how? J Clin Endocrinol Metab. (2015) 100:395–7. 10.1210/jc.2014-3766 25423573 PMC4318888

[86] Chan KC Lit LC Law EL Tai MH Yung CU Chan MH. Diminished urinary free cortisol excretion in patients with moderate and severe renal impairment. Clin Chem. (2004) 50:757–9. 10.1373/clinchem.2003.029934 15044334

[87] Kidambi S Raff H Findling JW. Limitations of nocturnal salivary cortisol and urine free cortisol in the diagnosis of mild Cushing's syndrome. Eur J Endocrinol. (2007) 157:725–31. 10.1530/EJE-07-0424 18057379

[88] Liu H Bravata DM Cabaccan J Raff H Ryzen E. Elevated late-night salivary cortisol levels in elderly male type 2 diabetic veterans. Clin Endocrinol. (2005) 63:642–9. 10.1111/j.1365-2265.2005.02395.x 16343098

[89] Butler P Besser G Pituitary-adrenal function in severe depressive illness. Lancet. (1968) 291:1234–6. 10.1016/S0140-6736(68)91927-2 4172780

[90] Badrick E Kirschbaum C Kumari M. The relationship between smoking status and cortisol secretion. J Clin Endocrinol Metab. (2007) 92:819–24. 10.1210/jc.2006-2155 17179195

[91] Nickelsen T Lissner W Schöffling K The dexamethasone suppression test and long-term contraceptive treatment: measurement of ACTH or salivary cortisol does not improve the reliability of the test. Exp Clin Endocrinol Diabetes. (1989) 94:275–80. 10.1055/s-0029-1210910 2560985

[92] Baid SK Sinaii N Wade M Rubino D Nieman LK. Radioimmunoassay and tandem mass spectrometry measurement of bedtime salivary cortisol levels: a comparison of assays to establish hypercortisolism. J Clin Endocrinol Metab. (2007) 92:3102–7. 10.1210/jc.2006-2861 17550962

[93] Elamin MB Murad MH Mullan R Erickson D Harris K Nadeem S. Accuracy of diagnostic tests for Cushing's syndrome: a systematic review and metaanalyses. J Clin Endocrinol Metab. (2008) 93:1553–62. 10.1210/jc.2008-0139 18334594

[94] Meikle AW. Dexamethasone suppression tests: usefulness of simultaneous measurement of plasma cortisol and dexamethasone. Clin Endocrinol. (1982) 16:401–8. 10.1111/j.1365-2265.1982.tb00733.x 7094363

[95] Mericq MV Cutler GB Jr. High fluid intake increases urine free cortisol excretion in normal subjects. J Clin Endocrinol Metab. (1998) 83:682–4. 10.1210/jcem.83.2.4555 9467592

[96] Briegel J Sprung CL Annane D Singer M Keh D Moreno R. Multicenter comparison of cortisol as measured by different methods in samples of patients with septic shock. Intensive Care Med. (2009) 35:2151–6. 10.1007/s00134-009-1627-9 19760208

[97] Pecori Giraldi F Saccani A Cavagnini F. Assessment of ACTH assay variability: a multicenter study. Eur J Endocrinol. (2011) 164:505–12. 10.1530/EJE-10-0962 21252174

[98] Galeandro L Sieber-Ruckstuhl NS Riond B Hartnack S Hofmann-Lehmann R Reusch CE. Urinary corticoid concentrations measuredby 5 different immunoassays and gas chromatography-mass spectrometry in healthy dogs and dogs with hypercortisolism at home and in the hospital. J Vet Intern Med. (2014) 28:1433–41. 10.1111/jvim.12399 25040917 PMC4895583

[99] Miller R Plessow F Rauh M Groschl M Kirschbaum C. Comparison of salivary cortisol as measured by different immunoassays and tandem mass spectrometry. Psychoneuroendocrinology. (2013) 38:50–7. 10.1016/j.psyneuen.2012.04.019 22641005

[100] Klose M Lange M Rasmussen AK Skakkebaek NE Hilsted L Haug E. Factors influencing the adrenocorticotropin test: role of contemporary cortisol assays, body composition, and oral contraceptive agents. J Clin Endocrinol Metab. (2007) 92:1326–33. 10.1210/jc.2006-1791 17244781

[101] Turpeinen U Hamalainen E. Determination of cortisol in serum, saliva and urine. Best Pract Res Clin Endocrinol Metab. (2013) 27:795–801. 10.1016/j.beem.2013.10.008 24275191

[102] Friedman TC Ghods DE Shahinian HK Zachery L Shayesteh N Seasholtz S. High prevalence of normal tests assessing hypercortisolism in subjects with mild and episodic Cushing's syndrome suggests that the paradigm for diagnosis and exclusion of Cushing's syndrome requires multiple testing. Horm Metab Res. (2010) 42:874–81. 10.1055/s-0030-1263128 20803415 PMC2978784

[103] Velez DA Mayberg MR Ludlam WH. Cyclic Cushing syndrome: definitions and treatment implications. Neurosurg Focus. (2007) 23:E4; discussion Ea. 10.3171/foc.2007.23.3.5 17961029

[104] Wester VL Reincke M Koper JW van den Akker ELT Manenschijn L Berr CM. Scalp hair cortisol for diagnosis of Cushing's syndrome. Eur J Endocrinol. (2017) 176:695–703. 10.1530/EJE-16-0873 28289104

[105] Eisenhofer G Masjkur J Peitzsch M Di Dalmazi G Bidlingmaier M Gruber M. Plasma steroid metabolome profiling for diagnosis and subtyping patients with Cushing syndrome. Clin Chem. (2018) 64:586–96. 10.1373/clinchem.2017.282582 29208661

[106] Masjkur J Gruber M Peitzsch M Kaden D Di Dalmazi G Bidlingmaier M. Plasma steroid profiles in subclinical compared with overt adrenal cushing syndrome. J Clin Endocrinol Metab. (2019) 104:4331–40. 10.1210/jc.2018-02349 30977834

[107] Eisenhofer G Dekkers T Peitzsch M Dietz AS Bidlingmaier M Treitl M. Mass spectrometry-based adrenal and peripheral venous steroid profiling for subtyping primary aldosteronism. Clin Chem. (2016) 62:514–24. 10.1373/clinchem.2015.251199 26787761

